# Instant formation of horizontally ordered nanofibrous hydrogel films and direct investigation of peculiar neuronal cell behaviors atop

**DOI:** 10.1186/s40824-023-00344-3

**Published:** 2023-03-13

**Authors:** Jaeil Park, Thi Thuy Chau Nguyen, Su-Jin Lee, Sungrok Wang, Dongmi Heo, Dong-Hee Kang, Alexander Tipan-Quishpe, Won-June Lee, Jongwon Lee, Sung Yun Yang, Myung-Han Yoon

**Affiliations:** 1 School of Materials Science and Engineering, Gwangju Institute of Science and Technology (GIST), 123 Cheomdangwagi-Ro, Buk-Gu, Gwangju, 61005 Republic of Korea; 2 Department of Polymer Science and Engineering, Graduate School of Chungnam National University, 99 Daehak-Ro, Yuseong-Gu, Daejeon, 34134 Republic of Korea

**Keywords:** Nano-fibrous hydrogel, Hydrogel dispersion, Bar coating, Self-alignment, Neuronal culture

## Abstract

**Background:**

Hydrogels have been widely used in many research fields owing to optical transparency, good biocompatibility, tunable mechanical properties, etc. Unlike typical hydrogels in the form of an unstructured bulk material, we developed aqueous dispersions of fiber-shaped hydrogel structures with high stability under ambient conditions and their application to various types of transparent soft cell culture interfaces with anisotropic nanoscale topography.

**Method:**

Nanofibers based on the polyvinyl alcohol and polyacrylic acid mixture were prepared by electrospinning and hydrogelified to nano-fibrous hydrogels (nFHs) after thermal crosslinking and sulfuric acid treatment. By modifying various material surfaces with positively-charged polymers, negatively-charged superabsorbent nFHs could be selectively patterned by employing micro-contact printing or horizontally aligned by applying shear force with a wired bar coater.

**Results:**

The angular distribution of bar-coated nFHs was dramatically reduced to ± 20° along the applied shear direction unlike the drop-coated nFHs which exhibit random orientations. Next, various types of cells were cultured on top of transparent soft nFHs which showed good viability and attachment while their behaviors could be easily monitored by both upright and inverted optical microscopy. Particularly, neuronal lineage cells such as PC 12 cells and embryonic hippocampal neurons showed highly stretched morphology along the overall fiber orientation with aspect ratios ranging from 1 to 14. Furthermore, the resultant neurite outgrowth and migration behaviors could be effectively controlled by the horizontal orientation and the three-dimensional arrangement of underlying nFHs, respectively.

**Conclusion:**

We expect that surface modifications with transparent soft nFHs will be beneficial for various biological/biomedical studies such as fundamental cellular studies, neuronal/stem cell and/or organoid cultures, implantable probe/device coatings, etc.

**Supplementary Information:**

The online version contains supplementary material available at 10.1186/s40824-023-00344-3.

## Introduction

Hydrogels are three-dimensional polymeric networks that contain large quantities of water within their porous structure [1, 2]. Hydrogels have drawn much attention in biological/biomedical research fields due to their responses to various external stimuli such as pH [3–5], temperature [6–8], and light [6, 9], and their soft mechanical properties similar to human tissues/organs [10, 11]. In particular, the modulus and structure of hydrogels are relatively easy to modulate [12], providing many opportunities for preparing extracellular matrix (ECM) for artificial cell/tissue cultures [13, 14]. In tissue engineering, ECM has been recently discovered to comprise structural proteins, glycosaminoglycans, polysaccharides, adhesion proteins, and to significantly affect cell physiology according to its composition and structure [15, 16]. In particular, a mechanical modulus and structure equivalent to those of a target tissue/organ is the first point to consider when preparing ECM for tissue/organ regeneration. From this perspective, hydrogels are one of the best candidate materials for biomimetic artificial ECM. Hydrogels based on biocompatible polymers typically show good cell viability and favorable mechanical moduli ranging from 1 to 100 kPa, which covers those of most biological tissues [10, 17].

Many different strategies for fabricating artificial ECM-like hydrogels have been thoroughly studied. Fan and coworkers prepared an injectable porous hydrogel for in vivo tissue regeneration using a spray freezing technique [18]. The resulting microparticle-annealed nano-fibrous hydrogel closely mimicked the interconnected microporous structure of the native ECM. Recently, micro-structured hydrogel scaffolds with anisotropic patterns have also been developed to regenerate highly ordered tissues composed of muscles, neurons, and so on. For instance, Vogel and coworkers reported a simple methodology to prepare anisotropic scaffolds [19]; they applied heating and uni-directional mechanical force to an isotropic fiber mesh, significantly decreasing the directional distribution of fibers and forming an anisotropic fiber mesh. Furthermore, Gomes and coworkers reported that an injectable anisotropic hydrogel composite could be prepared with aligned nanoparticles under a magnetic field and employed as an anisotropic cell culture scaffold [20]. Fu and coworkers developed highly biocompatible bioinks based on hybrid hydrogel microparticles for 3D printing of cell culture scaffolds [21]. They directly printed a series of biomimetic scaffolds with very high aspect ratios which support the growth of bone-marrow-derived mesenchymal stem cells and formation of cell spheroids. Nonetheless, the abovementioned hydrogels exist as a bulk material once crosslinked. In contrast, dispersions of nanostructured hydrogels, if any, could be widely utilized to modify the surface properties of existing substrates by coating them with transparent soft hydrogel nanostructures; this could be beneficial for a variety of fundamental biological studies as well as practical biomedical applications.

In this study, we developed, for the first time, a nano-fibrous hydrogel (nFH) dispersion using superabsorbent hydrogel nanofibers based on polyvinyl alcohol (PVA) and polyacrylic acid (PAA). Regardless of material types and curvatures, various material surfaces could be coated with as-prepared nFH. To achieve the strong adhesion of nFH to the underlying substrate, we utilized the layer-by-layer deposition technique using poly-L-lysine, a positively charged polymer that can electrostatically interact with negatively charged nFHs. We then investigated the directional distribution and spatial arrangement of drop- or bar-coated nFHs in response to varied shear forces exerted by different bar-coating conditions and nFH dispersion concentrations. Finally, neuronal cells were cultured on nFH-coated substrates, and their peculiar behaviors such as neurite outgrowth and cell migration, were thoroughly investigated as a function of nFH alignment and coverage.

## Results

### Hydrogelification of electrospun PVA-PAA nanofibers

**Fig. 1:**
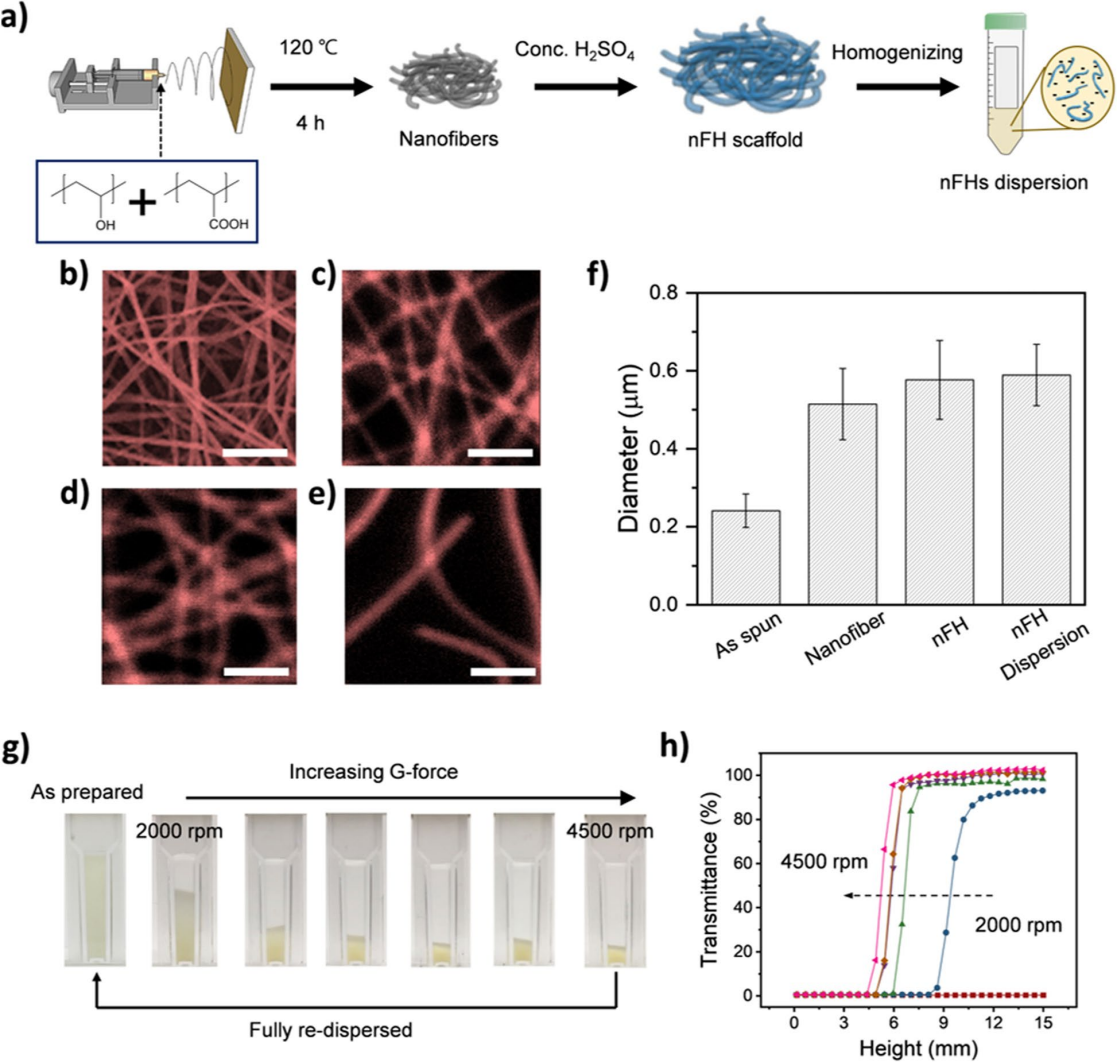
**a** Schematic illustration of preparing an nFH dispersion. **b** An scanning electron microscopy image of electrospun PVA-PAA nanofibers. Confocal microscopic image of **c** crosslinked PVA-PAA nanofibers, **d** nFHs, and **e**) homogenized nFHs. All scale bars denote 3 μm. **f** Diameters of nanofibers in b to e). **g** Photographic images of precipitated nFH dispersions after centrifugation. **h** Plots of optical transmittance of nFH dispersions after centrifugation as a function of height from the cuvette bottom. *g*-force was varied from 2000 to 4500 rpm with the increment of 500 rpm

A schematic procedure to prepare a nFH dispersion is displayed in Fig. 1a. Electrospun PVA-PAA nanofiber meshes were thermally annealed in an oven at 120 °C for 4 h for chemical crosslinking, followed by hydrogelification and homogenization (see the experimental for the details). The corresponding electron and optical microscopy images are shown in Fig. 1b-e. After homogenization, individual shortened nFHs are well dispersed in an aqueous medium. Furthermore, the overall density of hydrogel fibers in Fig. 1e is relatively lower than before homogenization at the same magnification. This indicates that an nFH dispersion can be easily diluted, whereas a mesh structure composed of long nFHs cannot. The diameters of electrospun nanofibers and nFHs were measured and summarized in Fig. 1f. The average diameter of electrospun nanofibers (Fig. 1b) was approximately 250 nm, and that of the crosslinked nanofiber (Fig. 1c) was 500 nm. As chemically crosslinked nanofibers were immersed in water for confocal laser scanning microscopy (CLSM) imaging, their diameters increased slightly due to swelling. After hydrogelification, their diameters increased further. With sulfuric acid treatment during the hydrogelification, a certain portion of hydroxyl groups are substituted by negatively charged sulfate groups [22, 23]. Then, nFH becomes more hydrophilic and absorbs more water molecules than before hydrogelification. Note that the average diameter of nFH was not affected by homogenization. The stability of an aqueous nFH dispersion was examined by centrifugation and re-dispersion. The aqueous nFH dispersion showed excellent stability at ambient conditions for more than six months, while the nFH dispersion could be concentrated by centrifuging at 2000 or higher rpm (Fig. 1g, h). The corresponding *g*-force values summarized in Table S1 confirms excellent long-term stability [24].

### Characteristics of nFH coatings

The stability of an aqueous nFH dispersion originates from negative charges in individual nFHs [25]. Therefore, an nFH dispersion was used as a coating solution on positively charged surfaces (Fig. S1). For instance, negatively-charged nFHs could be coated onto a glass substrate modified with positively-charged poly-L-lysine (PLL, Sigma Aldrich, MW 30,000 ~ 70,000) via electrostatic interaction. Although the substrate exhibited net negative charges, the PLL coating on the glass surface imparted partially positive charges (Fig. S1a). Note that if the glass substrate was coated with a more concentrated PLL solution, the substrate would have net positive charges [26, 27]. After the nFH dispersion was drop-coated on the PLL-modified surface, the surface zeta potential decreased to -31 mV, which suggests that negatively charged nFHs were adsorbed on the positively charged PLL layer by electrostatic interaction, which leads to the layer-by-layer assembly (Fig. S1b) [28, 29].

**Fig. 2:**
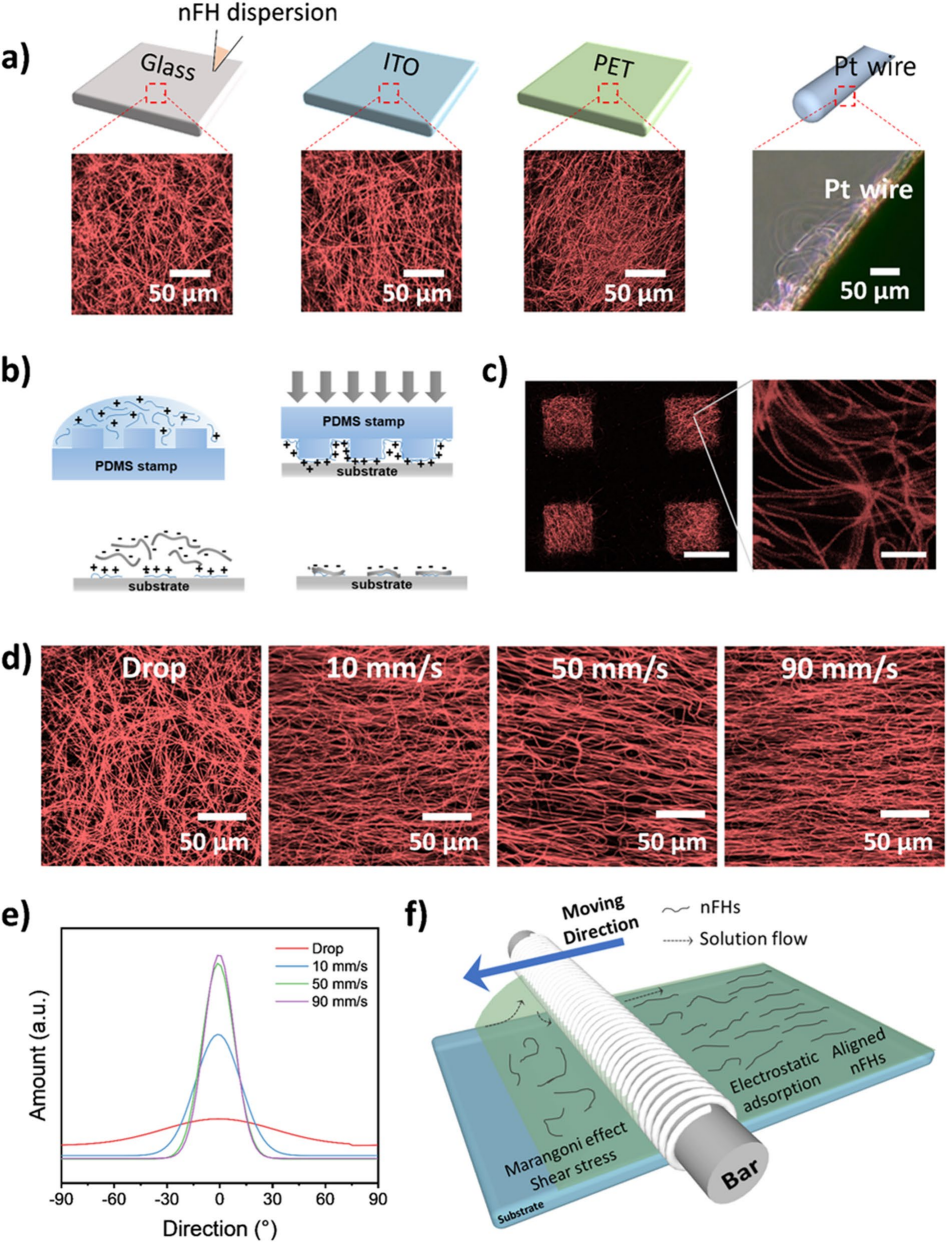
**a** Coating of of nFHs on flat glass, ITO, PET substrates, and a Pt wire. **b** Schematic illustration of nFH patterning with the micro-contact printing method. **c** Confocal microscopy images of patterned nFHs on a PLL-coated glass substrate. **d** Confocal microscopy images of drop- and bar-coated nFHs at designated bar-moving speeds. **e** Statistical distributions of nFHs shown in **d**. **f** Schematic illustration of aligning nFHs under shear forces

Next, we demonstrated that nFHs could be coated on various material substrates such as glass, indium tin oxide (ITO)-coated glass, polyethylene terephthalate (PET) film, and cylindrical Pt wire after pre-coating with a positively charged polymer layer (Fig. 2a) [30]. Furthermore, an arbitrary spatial pattern of nFHs could be easily formed on a flat surface by employing micro-contact printing. Once the positively charged PLL layer was patterned with the micro-contact printing method, the resultant substrate was covered with the nFH dispersion for 1 min, followed by rinsing. Note that the nFH layer was selectively deposited only on the positively charged PLL pattern, not on the negatively charged glass surface, via electrostatic interaction (Fig. 2b, c).

### Anisotropic coating of nFHs using a wired bar coater

The drop-coated nFHs showed a uniform but random distribution of nFHs, whereas the bar-coated nFHs showed a specific directionality parallel with the shear force direction (Fig. 2d). It is noteworthy that as the bar moved faster, larger shear force could be produced, and the resultant directional distribution of nFHs became narrower down to ± 20° (Fig. 2e). To our knowledge, this is the first demonstration of uni-directionally aligned hydrogel nanofibers. A schematic illustration of shear force generation and nFH aligning during the bar-coating is depicted in Fig. 2f. 1) Due to the hydrophilicity of a cylindrical metal bar wrapped with metallic wires, the aqueous nFH dispersion is attracted to the bar at the interface while the Marangoni effect produces a strong shear force. As a result, the coated nFHs showed a specific directional alignment in parallel with the shear force to reduce the resistance against the solution flow [31]. 2) The moving bar transfers its momentum to the nFHs by directly applying an additional shear force. 3) The bumped nFH flow under the bar and a weak shear force are produced by minimizing the Marangoni effect when the bar passes. 4) The aligned nFH layer is tethered on a PLL-coated substrate via electrostatic interaction.

**Fig. 3:**
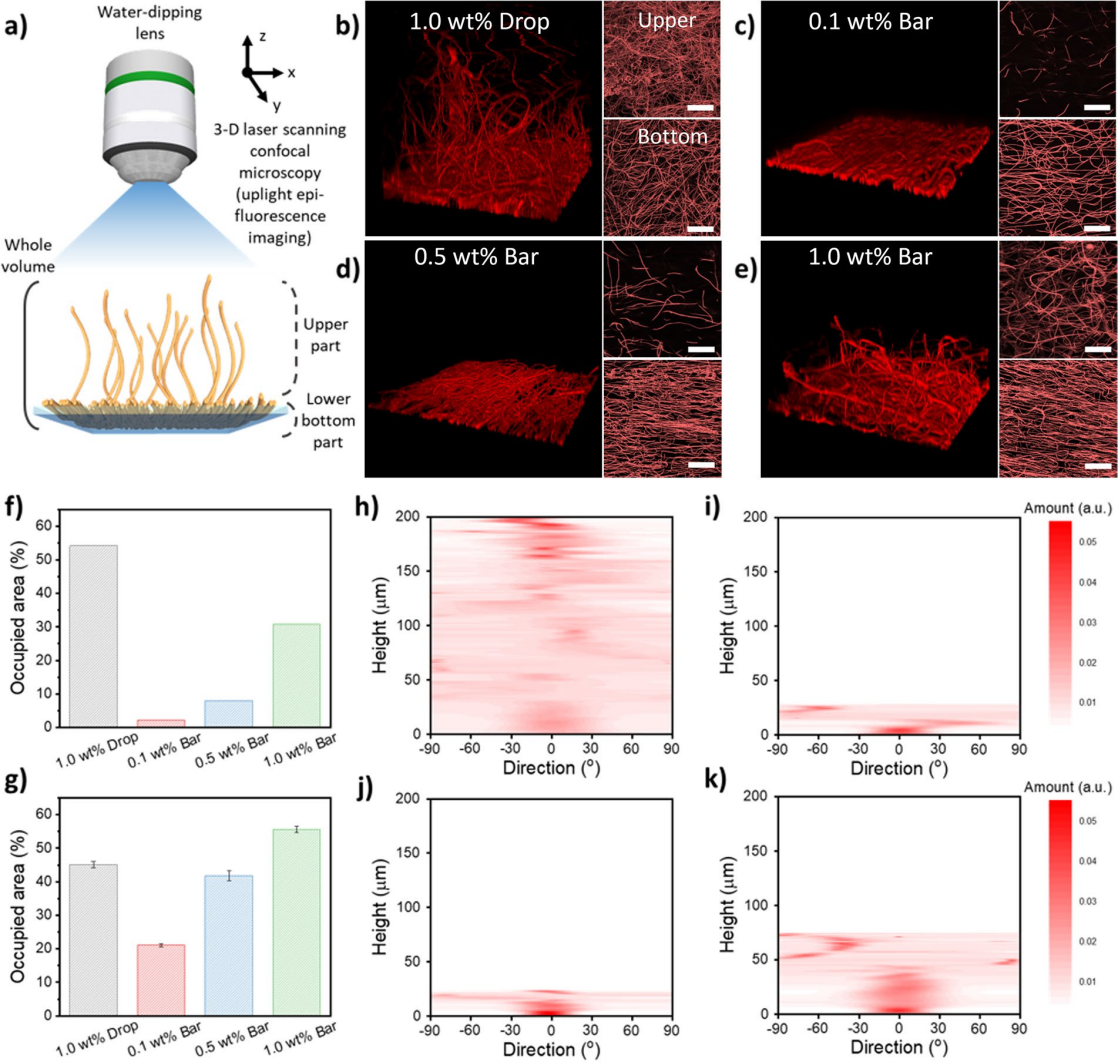
**a** Schematic illustration of upright confocal laser scanning microscopy of 3-D nFH arrangements with a water-dipping lens. CLSM images of **b** 1.0 wt% drop-coated, **c** 0.1 wt%, **d** 0.5 wt% and **e** 1.0 wt% bar-coated nFHs on glass substrates; 3-D-perspective reconstructed (left), upper-part (upper right) and lower-bottom-part (lower right) z-stack images of nFHs. Occupied area of nFHs **f** at the upper of a substrate and **g** at the bottom of a substrate, respectively. Z-stacked directionality of **h** 1.0 wt% drop coated, **i** 0.1 wt%, **j** 0.5 wt% and **k** 1.0 wt% bar coated nFHs on glass substrate

Before employing the nFH-aligned substrates for cell cultures, we performed the in-depth investigation of the three-dimensional (3-D) arrangements of coated nFHs depending on the nFH concentration (1 wt% drop-coated, 0.1, 0.5 and 1.0 wt% bar-coated). To examine the 3-D morphology of nFH in the wet condition, the bar-coated nFHs were appropriately stained and imaged using confocal microscopy instead of electron microscopy (Fig. 3a). Note that the 3-D reconstructed nFH images are displayed using the whole volume of vertically-scanned CLSM images (left), while the upper-part standing (upper right) and the lower-bottom-part adsorbed nFH images (lower right) are shown from the z-stacking perspective (Fig. 3b-e). Many standing nFHs could be observed at the upper layer of 1.0 wt% drop-coated substrate (Fig. 3b). It was clearly seen that the one end of nFHs were anchored at the surface with random horizontal orientations, while the other end of nFHs were standing again with random vertical orientations (Fig. S2). In the case of 0.1 and 0.5 wt% bar-coated nFH samples, however, most parts of nFHs were tethered on substrates with one or two layers of nFHs well aligned. This phenomenon is because, in the presence of sheer force, the relatively diluted nFH dispersion allows the substrate surface to be covered with aligned nFHs. Thus, most of the nFHs are adsorbed directly on the underlying substrate with a tiny number of nFHs protruding toward the upper part (Fig. 3c, d). But when the more concentrated (1.0 wt%) nFH dispersion was bar-coated, many randomly oriented nFHs were observed at the upper part similarly in the case of 1.0 wt% drop-coated substrate (Fig. 3e). Interestingly, the lower-bottom part close to the substrate surface showed well-aligned nFHs as in the case of 0.1 and 0.5 wt% bar-coated nFH samples (Fig. 3d). For more quantitative analysis, we extracted the occupied areas and directionality distributions of nFHs by processing both upper- and lower-bottom-part z-stacked LSCM images (Fig. 3f, g). The drop-coated sample showed the ~ 45 and 55% nFHs at the lower-bottom and upper parts, respectively. In contrast, bar-coated samples with diluted nFH dispersions (i.e., 0.1 and 0.5 wt%) showed substantially large occupied areas at the lower-bottom part (~ 20 and ~ 40%) than at the upper part (~ 2 and 8%). In the case of 1.0 wt% bar-coated substrate, this trend becomes less prominent, but the lower-bottom part (~ 55%) was still more occupied than the upper part (~ 30%). This is partly because when the bar passes a substrate, the excess nFH dispersion is wiped out and a lower volume of nFH dispersion is left on the substrate; thus, relatively less number of nFHs are remained. Furthermore, nFHs repel each other in an aqueous dispersion because of their excluded volumes and slight negative charges, resulting in the partial surface coverage by nFHs. Interestingly, the bar-coated 1.0 wt% sample exhibits larger occupied areas near the substrate surface than the drop-coated 1.0 wt% one. This result can be attributed to the height and directionality of bar-coated nFHs. As shown in Fig. 3h-k, nFHs on drop-coated substrate show 200 μm height of standings whereas bar-coated nFHs show only ~ 25 μm for 0.1 and 0.5 wt% and ~ 75 μm for 1.0 wt%. The nFHs on drop coated substrate show random directional distribution through whole z-stacked space. However, the lower parts of bar-coated nFHs show specific directional distributions (via applied shear force). Moreover, this trend becomes more pronounced and the resultant specific directionality enables more compact nFH deposition, thus, higher coverage on the surface with the increase of bar-coated nFH concentration. Note also that the upper parts of bar-coated nFHs show much less directionality than the lower-bottom parts but still relatively higher directionality than the upper parts of drop-coated nFHs (Fig. S3). This phenomenon can be explained by the partial locking of nFH directionality caused by the surface-tethered compact lower-bottom-part nFHs which exhibit the specific directionality and the consequent reduced motility of upper-part nFHs.

### Neuronal cell* (*PC12) cultures on nFH-coated substrates

**Fig. 4:**
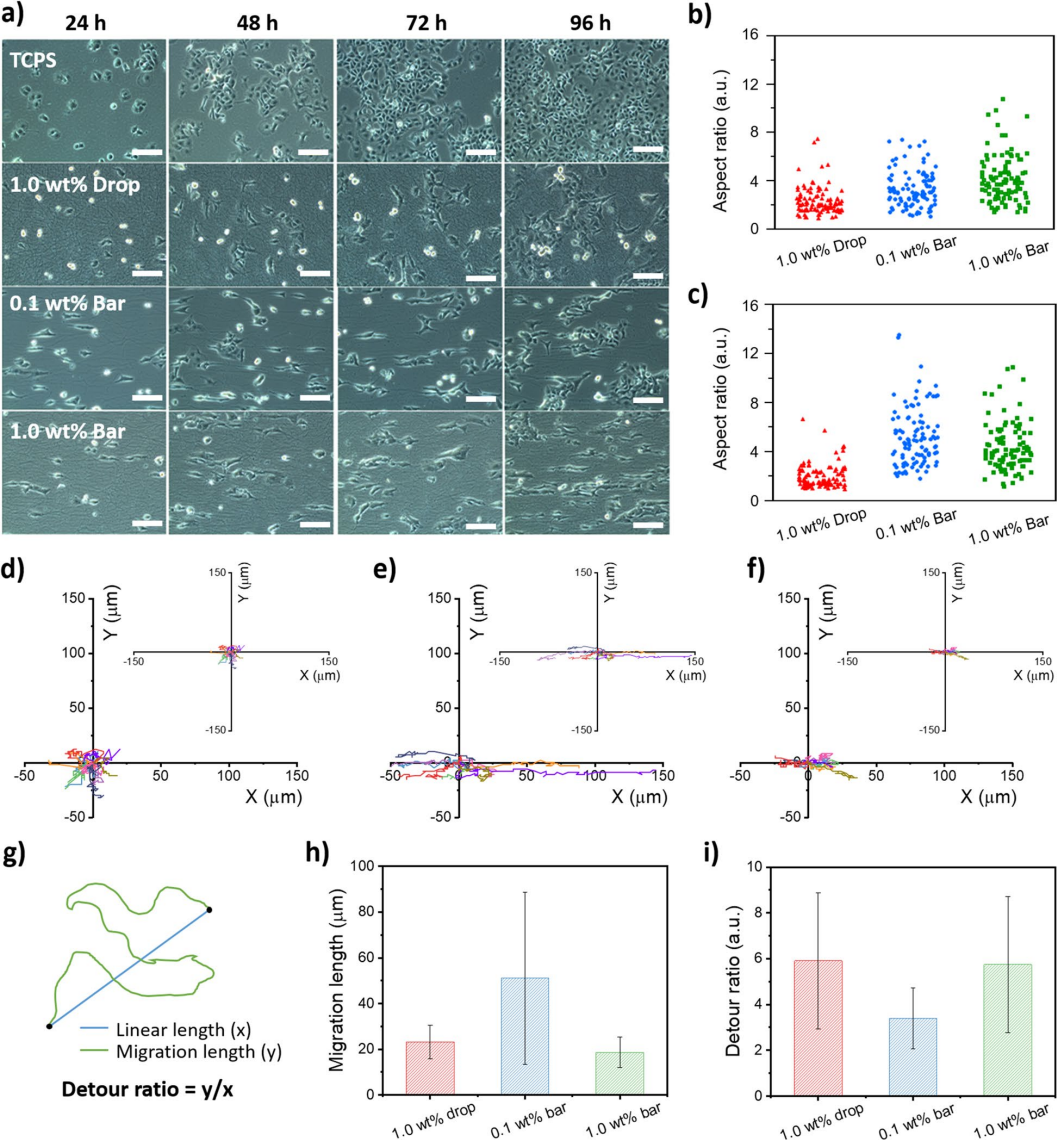
**a** Optical microscopy images of PC12 cells on nFH-coated surfaces observed for 96 h with 24 h intervals.; Tissue culture grade polystyrene (TCPS) bare surface, 1.0 wt% drop-coated, 0.1 wt% and 1.0 wt% bar-coated nFH surfaces (Scale bar, 100 μm). The aspect ratio of PC12 cells on nFH coated surfaces at **b** 24 h and **c** 72 h after cell seeding (*n* = 100). Trajectories of PC 12 cells cultured on **d** 1.0 wt% drop-coated, **e** 0.1 wt% and **f** 1.0 wt% bar-coated nFH substrates (see the experimental for the details). **g** Schematics of definitions of migration length and detour ratio. h Migration length and **i** detour ratio of PC 12 cells on three different substrates

First, cell viability was examined using the MTT assay, and the results are summarized in Fig. S4. All nFH-coated substrates supported good cell viability comparable to those on TCPS. In Fig. 4a, PC12 cells on TCPS appear circular or spike-edged square shapes, and the areas covered with proliferating cells are much closer to each other. This suggests that the PC12 cells remained locally as they initially attached to the surface without much movement. We observed similar phenomena with cells on the drop-coated nFH substrate for 24 h and no significant morphological changes were observed over time compared with those on TCPS. In contrast, the PC12 cells on bar-coated nFH substrates showed dramatic changes in their morphology. They were highly stretched along the overall aligned direction of underlying nFHs on both 0.1 and 1.0 wt% bar-coated samples; moreover, some of the stretched cells were connected to each other via dendrites.

For more quantitative analysis, we attempted to evaluate cell morphological changes by determining the aspect ratio (i.e., long-axis length/short-axis length; *l*_*a*_*/s*_*a*_) distributions extracted from microscopic cell images. At 24 h post-seeding, the PC12 cells on the drop-coated nFH substrate exhibited aspect ratios ranging from 1.0 to 7.5 with an average of 2.4 ± 1.1. Those on the 0.1 and 1.0 wt% bar-coated nFH substrates showed aspect ratios of 1.1 – 7.4 and 1.4 – 10.7 with an average of 3.4 ± 1.5 and 4.2 ± 1.9, respectively (Fig. 4b). Thus, PC12 cells were more stretched on aligned nFH-coated substrates while their aspect ratios were affected by the amount of deposited nFHs. The average aspect ratio values were similar regardless of the nFH concentration, but the upper-level values increased by approximately 2 times. As the occupied area of the 1.0 wt% bar-coated nFH is over 50% and that of 0.1 wt% bar-coated nFH is ~ 20%, more cells on the 1.0 wt% bar-coated nFH may only be in contact with nFHs. As they were exposed to more anisotropic environment, more highly stretched cells were observed. Although an nFH itself has an anisotropic fiber shape, the PC12 cells on the drop-coated nFH substrate were slightly stretched but still less stretched than those on bar-coated nFH substrates. Also, the upper *l*_*a*_*/s*_*a*_ limit of cells on the 0.1 wt% bar-coated nFH substrate increased from 7.4 to 13.5 as the culture time increased from 24 to 72 h (Fig. 4c). In cases of 1.0 wt% bar- and drop-coated nFH substrates, the *l*_*a*_*/s*_*a,*_ at 72 h did not change much from those at 24 h. Thus, PC12 cells stretched and interacted with each other most prominently on 0.1 wt% nFH with increasing time.

In parallel, we investigated the effect of 3D hydrogel fiber arrangements on cell motility by analyzing cell movement on nFH-coated substrates using time-lapse optical microscopy. We obtained and analyzed the time-lapse trajectories of PC12 cells cultured on 1.0 wt% drop-coated, 0.1 wt% and 1.0 wt% bar-coated nFH substrates. Cells on the drop-coated nFH showed the random moderate motility as represented by an average speed of 6.25 μm/h and non-specific radial directionality (Fig. 4d). In the case of the 0.1 wt% bar-coated nFH, most of cells traveled over 100 μm (max. 144.7 μm) with an average speed of 10.88 μm/h (Fig. 4e). More interestingly, their trajectories are pseudo one-dimensional (1-D), parallel with the average nFH orientation. In the case of the 1.0 wt% bar-coated nFH, we also observed the pseudo 1-D movement of cells atop but they showed significantly reduced motility as represented by the average travel distances of < 25 μm as well as an average speed of 4.95 μm/h (Fig. 4f). Maximum travel distances and average moving speeds of cells cultured on these substrates are summarized in Table S2. Note that the adhesion of cells to nFH-coated substrates could be attributed to the interaction between integrins on cell membranes and sulfate ester groups on nFHs [32]. When cells migrate, they continuously sense and follow sulfate groups along hydrogel fibers via receptor-mediated mechanosensing [33]. Therefore, cells on isotropically distributed nFHs prepared by drop-coating move randomly in all directions, while those on anisotropically aligned nFHs prepared by bar-coating move mainly along the direction of underlying nFHs. To understand the different cell motility between 0.1 and 1.0 wt% bar-coated nFH substrates, the statistics of cell migration length and detour ratio during the 12-h culture (see Fig. 4g for definitions) are also extracted. Cells cultured on the 0.1 wt% bar-coated nFH substrate showed the most extended migration length and shortest detour ratio (Fig. 4h, i). Note that the detour ratio indicates the extent to which a cell bypasses instead of moving forward persistently to the final position. Both 1.0 wt% drop- and bar-coated substrates, which exhibit a large number of entangled nFHs in the upper layer (Fig. 3e), reduce cell migration length and enhance cell detour probability. Based on this, we suppose that vertically protruding nFHs may physically block cell’s linear migration even in well-aligned nFHs underneath, which explains the relatively low travel length shown in Fig. 4f. In addition, a certain portion of nFHs may sit entangled on top of underlying random/aligned nFHs and form barriers to impede cell’s linear migration. To further investigate whether the elongated cell morphology involves the inheritable gene-level changes, the cells grown on nFH-coated substrates were harvested, transferred to TCPS, and re-cultured. As shown in Fig. S5, the cells which exhibited highly stretched morphology reverted to those with normal morphology on a flat TCPS surface. Therefore, we suppose that the elongated morphology on bar-coated nFH substrates is a dynamic response of the cell to the underlying aligned nFHs, not a result of any permanent change at the gene level. In parallel, the neurite outgrowth of PC12 cells was observed after adding nerve growth factors (NGF) and the results are summarized in Fig. S6. The cells cultured on a control substrate without NGF are relatively rounded and do not exhibit any visible neurites. However, the NGF treatment for up to 36 h resulted in neurite outgrowth in PC12 cells. In particular, PC12 cells on the 0.1 wt% bar-coated nFH substrate showed the most significant neurite development [34].

### Primary culture of rat embryonic hippocampal neurons on nFH-coated substrates

**Fig. 5:**
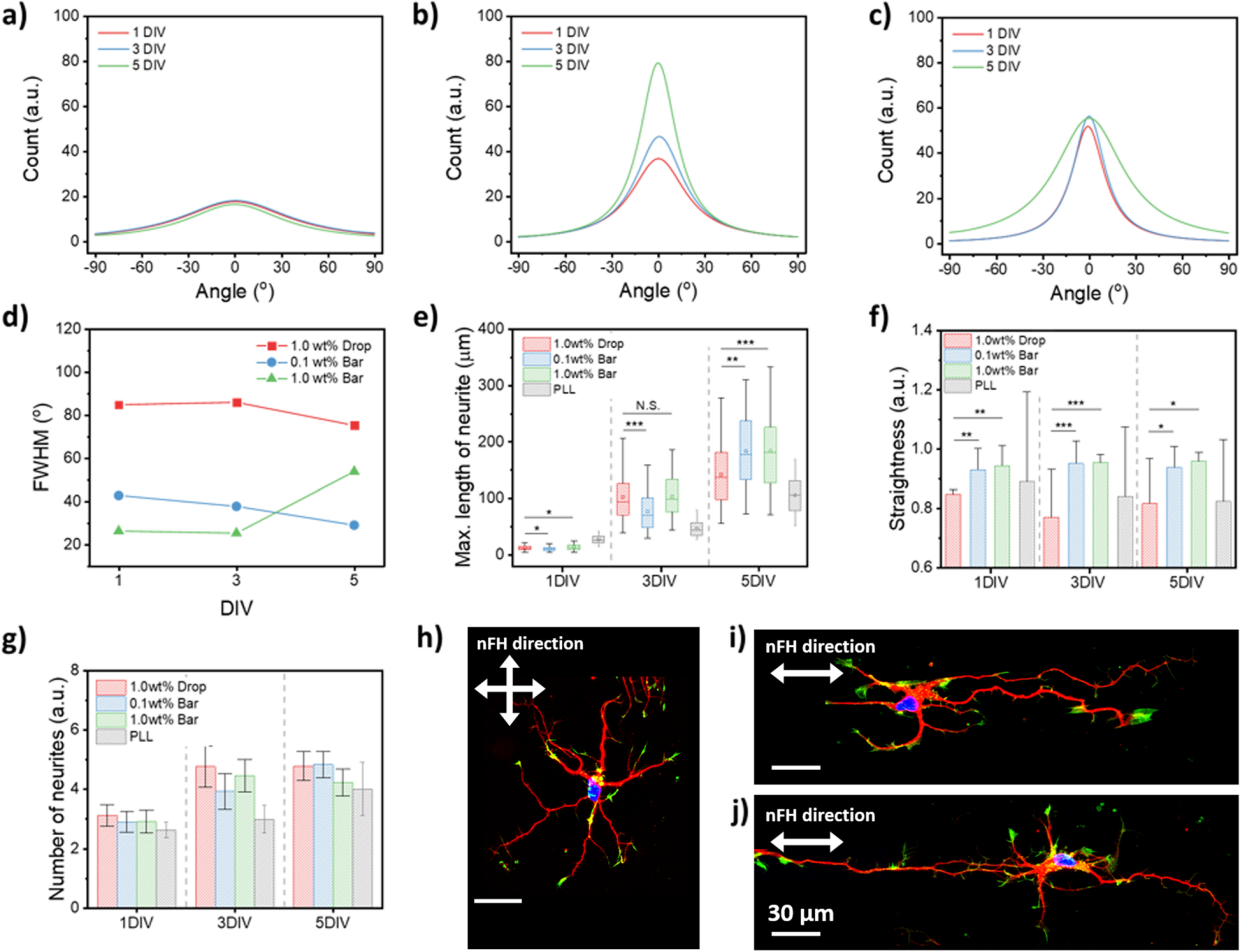
Directional distributions of neurites of primarily cultured embryonic (E18) rat hippocampal neurons on **a** 1.0 wt% nFHs drop coated, **b** 0.1 wt% nFHs bar coated, **c** 1.0 wt% nFHs bar coated glass surface. The direction of nFH on a glass substrate is set as 0°. Plots of **d** full-width at half-maximum (FWHM) of neurite directional distribution curves, **e** maximum neurite length, **f** neurite straightness, and **g** number of neurites sprout from each neuronal cell body as a function of days in vitro (DIV) of primary culture, depending on the surface modification type (PLL, 1.0wt% 0.1wt% bar-coated, 1.0wt% drop-coated nFHs). Individual groups were compared using Student’s t-test. ***, *P* ≤ 0.001; **, *P* ≤ 0.01; *, *P* ≤ 0.05, NS = non-significant. Representative immunofluorescence microscopic images of neuronal cells on **h** 1.0 wt% nFHs drop-coated, **i** 0.1 wt% nFHs bar-coated, **j** 1.0 wt% nFHs bar-coated glass surface at 5 DIV. Blue: nucleus, Red: tau, Green: actin

Next, we monitored the primary culture of rat embryonic hippocampal neurons on nFH- and only PLL-coated glass for comparison. Live and dead assay was carried out to examine cell viability and obtain other statistical data sets of neurons and neurites (Fig. S7). The cell viability on nFH-coated substrates was above 80% for 5 days in vitro (DIV) regardless of nanofiber directionality (Fig. S8). However, the cell viability on only PLL-coated substrates decreased to approximately 60% after 5 days. These results suggest that the positive charges of PLL might cause strong interactions with negatively charged cell surfaces, enhancing cell attachment but reducing cell movement, eventually reducing cell proliferation [35]. The positive charges of PLL were compensated by the negative charges from the nFH coating, which is verified in Fig. S1. Note that viability of neurons on 0.1 wt% bar-coated substrates, which positive charges of PLL may be partially compensated, gradually decreased from 95% at 1 DIV to 83% at 5 DIV whereas neurons on 1.0 wt% drop- and bar-coated substrates maintained the viability of approximately 90% at 5 DIV. Furthermore, we speculate that sulfate groups of nFH enhanced the adhesion of neuronal cells onto underlying substrates, resulting in decreasing detachment-induced apoptosis [35]. However, the directionality of neurites is highly dependent on the concentration and directionality of coated nFHs. On 1.0 wt% drop-coated substrates, where the directions of nFHs are uniformly distributed, neurites were also randomly spread out (Fig. 5a). On the other hand, the directions of neurites on 0.1 wt% and 1.0 wt% bar-coated substrates showed much narrower distributions as underlying nFHs became more aligned (Fig. 5b, c). Interestingly, the full-width at half maximum (FWHM) of the directional distribution of neurites on 0.1 wt% bar-coated substrates gradually decreased whereas that on 1.0 wt% bar-coated substrates increased. Note that the FWHM is a quantitative metric that evaluates how wide the directional distribution is. On 0.1 wt% bar-coated substrates, nFHs are sufficiently apart due to their low density and neurites grow along a single or a few underlying nFHs. On 1.0 wt% bar-coated substrates, however, nFHs are sufficiently close to each other due to their high density and neurite outgrowth are not limited to a single or a few underlying nFHs, resulting in the large FWHM (Fig. 5d). The maximum neurite length on drop-coated nFH substrates is not significantly different from that on bar-coated nFH substrates until 3 DIV. However, neuronal cells cultured on bar-coated nFH substrates exhibited longer neurites than those on drop-coated nFH substrates from 5 DIV (Fig. 5e). The neurite straightness of neurites on bar-coated nFH substrates is more prominent than that on drop-coated nFH substrates even at 1 DIV, regardless of nFH concentration employed for bar coating (Fig. 5f). These results indicate that the anisotropic nature of bar-coated nFHs induce more elongated as well as straight neurite outgrowth. It is also noteworthy that, unlike neurite elongation and straightness, the number of neurites was not significantly affected by the concentration and directionality of nFH (Fig. 5g). The similar behaviors of primarily cultured neurons were observed in the presence of surface nanotopography for contact cues [36]. The representative microscopic images of immunostained neuronal cells show that while neurites on the 1.0 wt% drop-coated nFH substrate are stretched with random orientations (Fig. 5h), those on 0.1 and 1.0 wt% bar-coated nFH substrates are aligned in an uni-directional orientation which is parallel to the overall nanofiber direction (Fig. 5i, j). Note also that despite many hydrogel fibers standing on the 1.0 wt% drop-coated nFH substrate, neurites are possibly settled down to the surface of aligned nFHs, because floating nFHs are sparsely located while they are continuously moving in a culture media.

## Discussion

ECM affects the mechanical modulus and overall 3D arrangement of a given tissue/organ while different cells exhibit distinct morphologies depending on their functions. For example, muscle cells exhibit typically stretched shapes which facilitate contraction/relaxation movements. Therefore, in addition to soft surfaces, anisotropic patterns which are known to be effective for inducing relevant cell morphology are essential in emulating physiological functions and properties which belong to a specific type of cell such as muscle cells, cardiomyocytes, neurons, etc. The aim of this study is preparing a dispersion of transparent soft nanofibrous hydrogels (nFHs) and fabricating nFH coating-based cell culture interfaces using a simple mass-producible method targeting at an artificial ECM which effectively control cell behaviors (e.g., morphology, motility, functions) atop.

The proposed nFHs show several distinct features as follows. First, nFH dispersions show excellent long-term stability since the repulsion between negatively charged nFHs is stronger than the gravitational force (Fig. 1g, h). Indeed, as very high centrifugal force is applied, the repulsion between negatively charged nFHs is overcome and the nFH dispersion is concentrated. Remarkably, the concentrated nFHs could be fully re-dispersed in water. This result is consistent with the observation that highly concentrated (up to 5 wt%) nFH dispersions are easily re-diluted without precipitation. Second, negatively charged nFH could be coated on various material substrates, for example, glass, ITO-coated glass, PET film, and Pt wire via layer-by-layer assembly using a positively charged polymer interlayer (Fig. 2a). Furthermore, in principle, arbitrary geometric patterns (e.g., lines, circles, squares, etc.) can be easily fabricated by preparing the mold for micro-contact printing. Third, most importantly, nFHs can be deposited with a specific directionality in parallel with the shear force direction using a wired bar coater (Fig. 2d). Nonetheless, nFH heads are freely standing while the majority of NFH bodies are attached on the surface (Fig. 3b-e).

In the next stage, we demonstrated that nFH could be beneficial for culturing immortalized cell lines as well as primary neuronal cells and investigating cell morphology and motility. First of all, transparent soft nFHs support cell viability/attachment atop as well as enable both inverted and upright optical microscopy of cultured cells. Furthermore, anisotropic fiber shapes induce stretched cell morphology and permit cell motility modulation, depending on their deposition conditions, in particular, drop- vs. bar-coating and nFH ink concentration. Interestingly, it was verified that the elongated cell morphology on bar-coated nFHs is a dynamic cellular response to the anisotropically aligned hydrogel fibers, but this trait is not inherited at the gene level (Fig. S5). More remarkably, we observed that bar-coated aligned nFHs induce more elongated and more straight neurite outgrowth than drop-coated randomly-oriented nFHs. All these results prove the versatility of nFHs for cellular behavior modulation and we expect that surface modifications with transparent soft nFHs will significantly contribute to many interdisciplinary research fields such as fundamental cellular studies, stem cell and/or organoid cultures, implantable probe/device coating development, etc. Currently, we are attempting to prepare 3-D anisotropic cell culture scaffolds using our nFH dispersions and their multiple coatings so that more realistic 3-D culture environments can be realized which mimic physiological conditions more closely.

## Conclusions

In this research, we developed a fibrous hydrogel dispersion as a coating solution and investigated cell morphology and motility on nFH-modified substrates. While nFH dispersions showed good stability for six months under ambient conditions, nFHs could be coated on the surfaces of many different materials such as glass, ITO, PET, and Pt wire via layer-by-layer assembly with positively charged polymers (e.g., PLL). Furthermore, hydrogel nanofibers could be aligned on the surface by applying shear forces using a wired bar coater. The bar-coated nFH exhibited a specific directional distribution parallel to the shear direction, whereas the drop-coated nFH showed uniform distributions in all directions. Furthermore, laser scanning confocal microscopy confirmed that, in the case of 1.0 wt% bar-coated nFHs, the tails of hydrogel nanofibers are vertically standing when the surface are compactly covered with aligned nFHs. Primarily cultured neuronal cells on bar-coated nFH substrates showed highly-stretched neurites with aspect ratios ranging from 1 to 14, while the vertical tails of hydrogel nanofibers impede the migration of neuronal cells cultured atop. Therefore, we expect that the nFH-modified substrates will serve as a versatile platform for fundamental cellular studies as well as bioelectric device development.

## Methods

### Electrospinning and hydrogelification of PVA-PAA nanofibers

For electrospinning, an aqueous solution of 8 wt% PVA-PAA (PVA:PAA = 8:2, solid content ratio) was prepared and PVA-PAA nanofibers were electrospun for 2 h using a 23G needle at a feed rate of 0.4 mL/hr. A voltage of 17 kV was applied while the distance between the needle and collector was set 13 cm and the collector drum covered with aluminum foil was rotated at 10 rpm. Next, the electrospun nanofiber (NF) mesh was annealed for crosslinking at 120 °C for 4 h. Subsequently, the crosslinked nanofiber mesh was immersed into concentrated sulfuric acid (conc-H_2_SO_4_). After ~ 3 s, the opaque nanofiber mesh was transformed into a transparent hydrogel sheet, rinsed with DI water several times, and stored in DI water. After one day, the hydrogel sheet in excess water was fragmented into fibrous hydrogel using a homogenizer (HG-15A, DAEHAN Scientific). Finally, the nFH dispersion was filtered through sieves with 400 μm, 200 μm, 100 μm, and 50 μm pores, successively, and the filtered nFH dispersion was concentrated to ~ 5 wt% by centrifugation before use.

### Coating substrates with the nFH dispersion

All coating processes were performed at room temperature under ambient conditions. Each substrate was cleaned and treated with oxygen plasma for 1 min at an intensity of 100 W (CUTE, Femto Science). Next, the substrate was covered with the diluted PLL solution (0.1 wt%) for 5 min, followed by rinsing and drying. For drop coating, PLL-modified substrates were covered with the diluted nFH dispersion for 5 min. For bar coating, the diluted nFH dispersion was dispensed only at one end of PLL-coated substrates. Then, the wire-wound bar was moved horizontally at a constant pulling rate of the nFH dispersion, while the constant distance between the bar and substrate was maintained at ~ 100 μm during the bar-coating process. To pattern the nFH layer on a substrate, PLL was patterned using the micro-contact printing method. A 1 wt% PLL aqueous solution was dropped on the PDMS stamp with a particular pattern, incubated at room temperature for 5 min, and rinsed with DI water. Subsequently, the oxygen plasma-treated glass substrate was contacted with the PLL-coated PDMS stamp for 30 s and covered with the nFH dispersion for 1 min after the stamp removal.

### PC12 cell culture and cell morphology analysis

PC12 cells (Korean Cell Line Bank) were cultured in the medium of RPMI-1640 (Gibco) supplemented with 10% fetal bovine serum (Gibco), 10 U mL^–1^ penicillin, and 10 mg mL^–1^ streptomycin (Gibco) under standard cell culture conditions (37 °C and 5% CO_2_). PC12 cells were seeded onto nFH-coated substrates at a density of 1.0 × 10^4^ cells cm^–2^. For cell viability test, the MTT assay was performed with the cells cultured on nFH-coated substrates for 2 days of in vitro (2 DIV) culture. After seeding cells, cell morphology was monitored and imaged using an optical microscope (IX71, Olympus). The images of > 100 cells were processed with Image J software to calculate the image cells' aspect ratios (long axis length/short axis width). To check the effect of nFHs on PC12 cell morphology at a genetic level, the cells were harvested with trypsin and transferred to TCPS plates after the 96 h-culture on nFH-modified substrates. NGF was added in the cell culture medium with a concentration of 50 ng/ml after 12 h of cell seeding to ensure providing sufficient time for cell attachment on the substrate. Neurite development was monitored up to 36 h, and processed with Image J software for the statistical analysis.

### Live cell imaging and cell migration analysis

The cell migration assay was conducted to assess the motility of cells cultured on nFH-coated substrates. PC12 cells were seeded on nFH-coated substrates surface at a density of 2.0 × 10^4^ cells cm^–2^, and bright- field images of cells were acquired with an optical microscope equipped with the environment-controlled chamber (Chamlide TC-L-Z003, Live Cell Instrument, South Korea). The cell images were captured at 10-min intervals after 30 min to 24 h after seeding, and the collected images were converted to movie files. Ten representative individual centroids of cells were collected from each sample and tracked manually from the obtained movie files using Image J software. The starting point of each cell was translocated to the origin in the trajectory plot to compare the migration trends of cells.

### Primary culture of embryonic hippocampal neurons

All samples were sterilized prior to cell culture by soaking the sample of 1% penicillin/streptomycin (10,000 units/ml, Invitrogen) and added DMEM (Dulbecco’s Modified Eagle Medium containing high glucose and pyruvate, Gibco). First, Sprague Dawley rat which was pregnant 18 days (E18) was euthanized with carbon dioxide gas. Then, surgical scissors and forceps were used to open the abdomen of the pregnant rat and uterus was taken out. Next, the embryos were collected and decapitated by a small surgical scissor. The brains were dissected from the heads of the embryos and the hippocampi was collected. Finally, the embryonic hippocampi were collected in 10 ml of HBSS (Hanks’ Balanced Salt Solution, Sigma Aldrich) containing 1% penicillin/streptomycin and 10 mM HEPES (4-(2-hydroxyethyl)-1-piperazineethanesulfonic acid, Sigma Aldrich). The collected hippocampi were transferred to clean bench and washed by HBSS three times, and incubated in papain solution which made of 160 µl of papain suspension (Worthington Biochem. Corp.), 30 ml of DNase 1 (Sigma Aldrich), and 10 ml of HBSS for 20 min at 37 ℃ and 5% CO_2_. After incubating hippocampi in papain solution, the supernatant was removed until 2 ml left and 10% FBS with DMEM was added to deactivate residual papain. And the hippocampi were gently triturated by mild pipetting with 30 μL DNase in 2 mL of HBSS. The number of cells was calculated by cell counter (Luna II automated cell counter) and it was diluted with plating medium which made of 10% FBS and HEPES-buffered DMEM. All the samples were washed with by PBS (Gibco) before cell seeding, and 4 × 104 cells were seeded on each sample. After 30 min plating, 20 mL of neurobasal media (Gibco) was poured. All the procedures for animal experiments have been approved by the Gwangju Institute of Science and Technology's the Institutional Animal Care and Use Committee.

### Immunostaining of neuronal cell on a nFH coated substrate

To analyze the growth of neurons, 1, 3, 5 days in vitro (DIV) were chosen for immunostaining. For immunostaining of cells, 4% (w/v) paraformaldehyde and 0.025% Triton X-100 (Sigma-Aldrich) in PBS were used for fixation and permeabilization of neurons on nFH and PLL-coated glass. For the passivation of cells, 10% BSA in PBS used. To visualize actin filaments and axons in neurons, phalloidin (Invitrogen) and mouse monoclonal anti-Tau-1 antibody (Santa Cruz Biotech. USA) were used together. Goat anti-mouse IgG antibody was used as a secondary antibody, and each toxin and antibody were used with Alexa Fluor Dye-labeled products (Invitrogen). The morphology of neurons was observed using upright confocal microscope (Olympus FV1000). The images were obtained using 20X water-dipping lens (XLUMPLFLN-W, Olympus) and 60 × water-dipping lens (UMPLFLN-W).

## Supplementary Information

**Additional file 1.**

## Data Availability

All relevant data are available within the article and its supplementary information files, or available from the corresponding authors upon reasonable request.

## Abbreviations

PVA: Polyvinyl alcohol
PAA: Polyacrylic acid
PLL: Poly-L-lysine
NF: Nanofiber
NFH: Nano-fibrous hydrogel
TCPS: Tissue culture grade polystyrene
PDMS: Polydimethylsiloxane
ITO: Indium tin oxide
PET: Polyethylene terephthalate
ECM: Extra cellular matrix
CLSM: Confocal laser scanning microscopy
FWHM: Full width at half maximum
DIV: Days in vitro
