# Supplementary material for: Instant formation of horizontally ordered nanofibrous hydrogel films and direct investigation of peculiar neuronal cell behaviors atop
Source: Biomater Res. 2023 Mar 13;27:19. doi: 10.1186/s40824-023-00344-3 (PMC10009932; doi:10.1186/s40824-023-00344-3)
Supplement: Supplementary file 1 — Additional file 1. [file 40824_2023_344_MOESM1_ESM.docx]

***Supplementary Information***

**Instant Formation of Horizontally Ordered Nanofibrous Hydrogel Films and Direct Investigation of Peculiar Neuronal Cell Behaviors Atop**

Jaeil Park,^a§^ Thi Thuy Chau Nguyen,^b§^ Su-Jin Lee,^b^ Sungrok Wang,^a^ Dongmi Heo,^a^ Dong-Hee Kang,^a^ Alexander Tipan-Quishpe,^a^ Won-June Lee,^a^ Jongwon Lee,^a^ Sung Yun Yang,^b,*^ and Myung-Han Yoon^a,*^

*^a^**School of Materials Science and Engineering, Gwangju Institute of Science and Technology (GIST), 123 Cheomdangwagi-ro, Buk-gu, Gwangju 61005, Republic of Korea,*

*^b^**Department of Polymer Science and Engineering, Graduate School of Chungnam National University, 99 Daehak-Ro, Yuseong-gu, Daejeon 34134, Republic of Korea*

^§^These authors contributed equally to this work

*Corresponding authors: Professor Sung Yun Yang and Professor Myung-Han Yoon

E-mail: sungyun@cnu.ac.kr and mhyoon@gist.ac.kr

**Table S1.** Conversion table of RPM to *g*-force with the rotational radius of 12.5 cm

| RPM | 2000 | 2500 | 3000 | 3500 | 4000 | 4500 |
| --- | --- | --- | --- | --- | --- | --- |
| *g*-force | 560 | 875 | 1260 | 1715 | 2240 | 2835 |

**
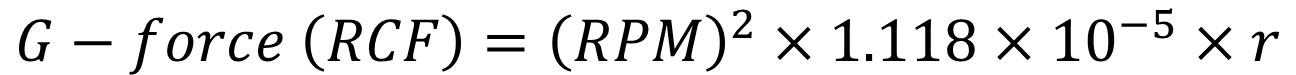
**

**Table S2.** Maximum moving distances and average moving speeds of PC12 cells cultured on various nFH-coated substrates.

| Sample | Max. moving distance (μm) | Ave. moving speed  (μm/h) |
| --- | --- | --- |
| 1.0 wt% drop-coated | 32.22 | 6.25 |
| 0.1 wt% bar-coated | 144.90 | 10.88 |
| 1.0 wt% bar-coated | 34.17 | 4.95 |


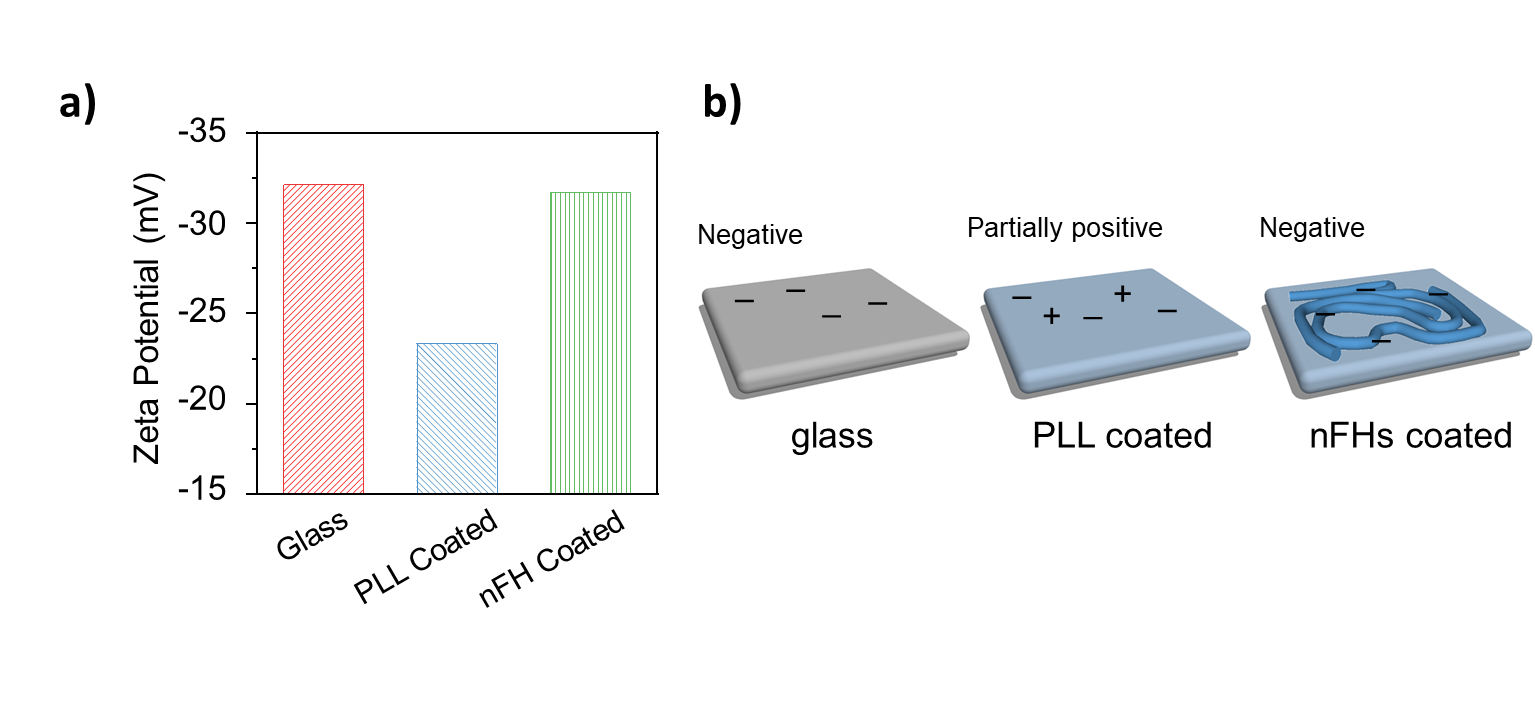


**Figure S1.** a) Plots of surface zeta potentials and b) Schemes of bare, PLL-coated, and PLL/nFH-coated glass substrates.


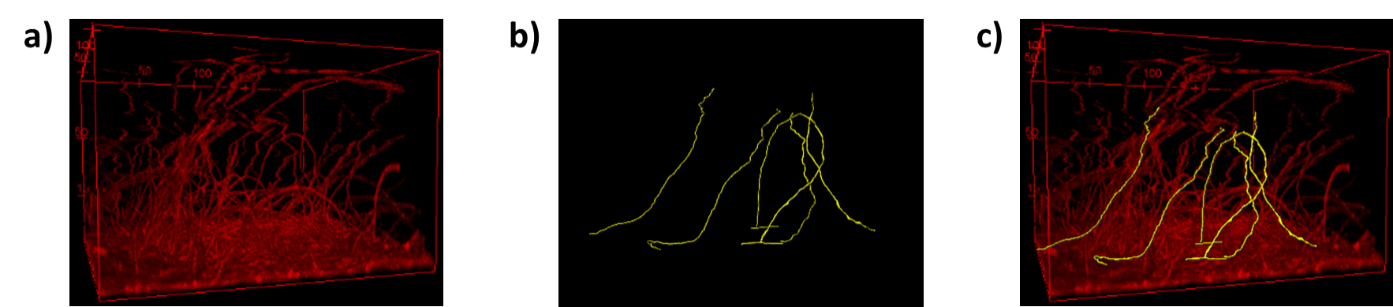


**Figure S2.** a) 3-D-perspective reconstructed CSLM image of the drop-coated nFH substrate. b) Images of three representative tracked nFHs. c) Overlay of a) and b).


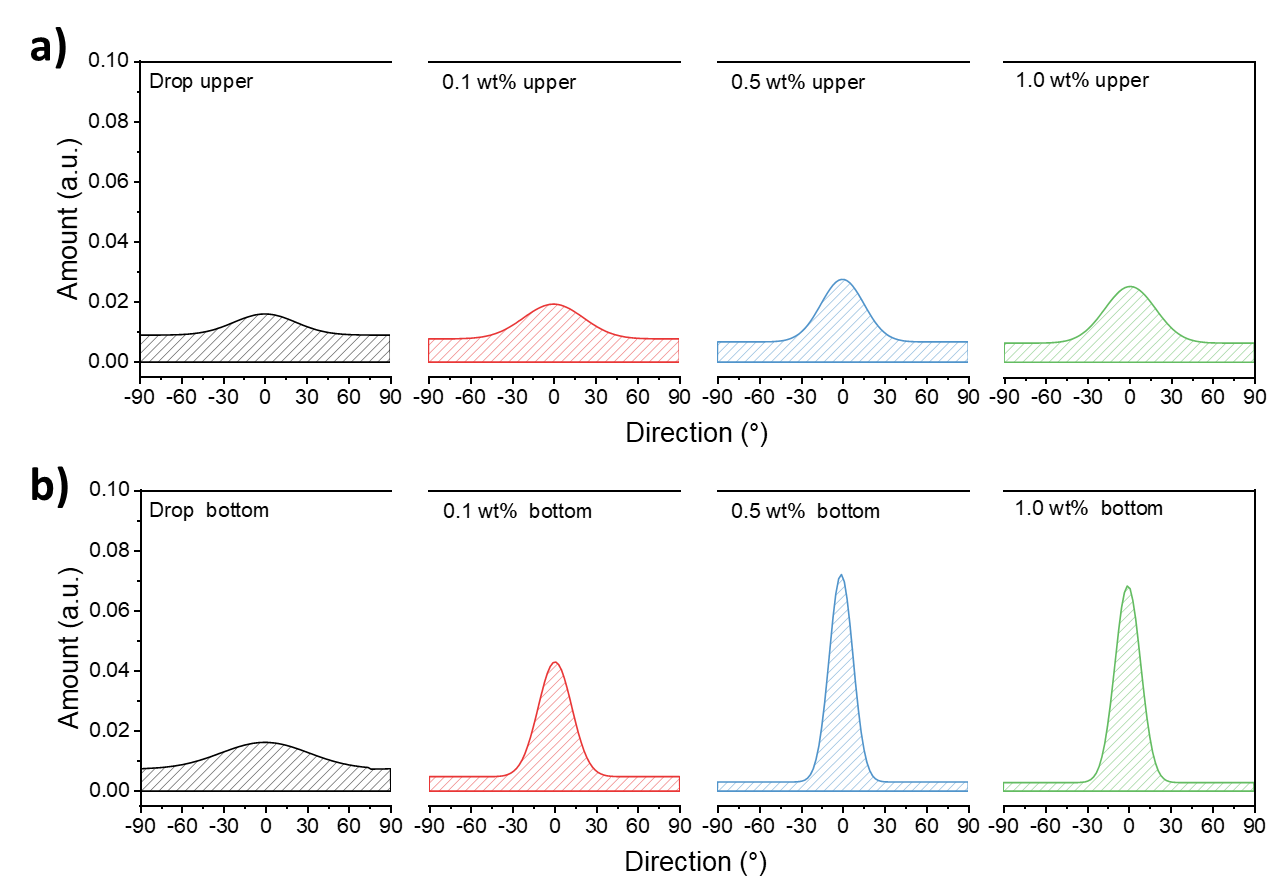


**Figure S3**. Directional distribution of nFHs a) at upper of a substrate and b) at bottom of a substrate respectively.


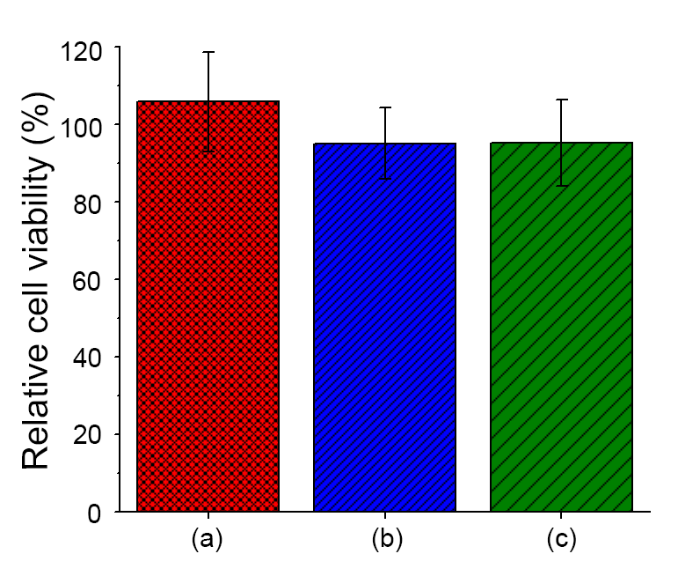


**Figure S4**. Relative cell viability of PC12 cells cultured on a) 1.0 wt% drop-coated, b) 0.1 wt% and c) 1.0 wt% bar-coated nFH substrates normalized with those on TCPS.


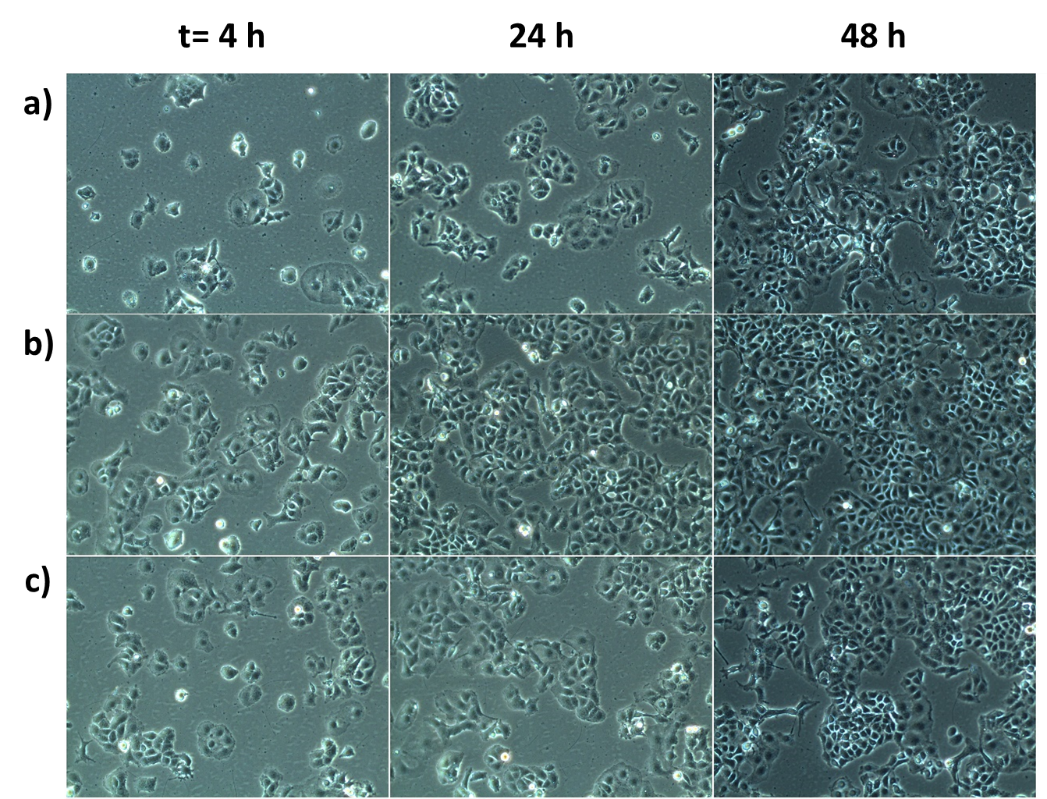


**Figure S5.** Optical microscopy images of PC12 cells transferred to TCPS after culturing on a) 1.0 wt% drop-coated, b) 0.1 wt% and c) 1.0 wt% bar-coated nFH substrates for 96 h. ‘t’ represents the time elapsed after transfer to TCPS and re-culture.


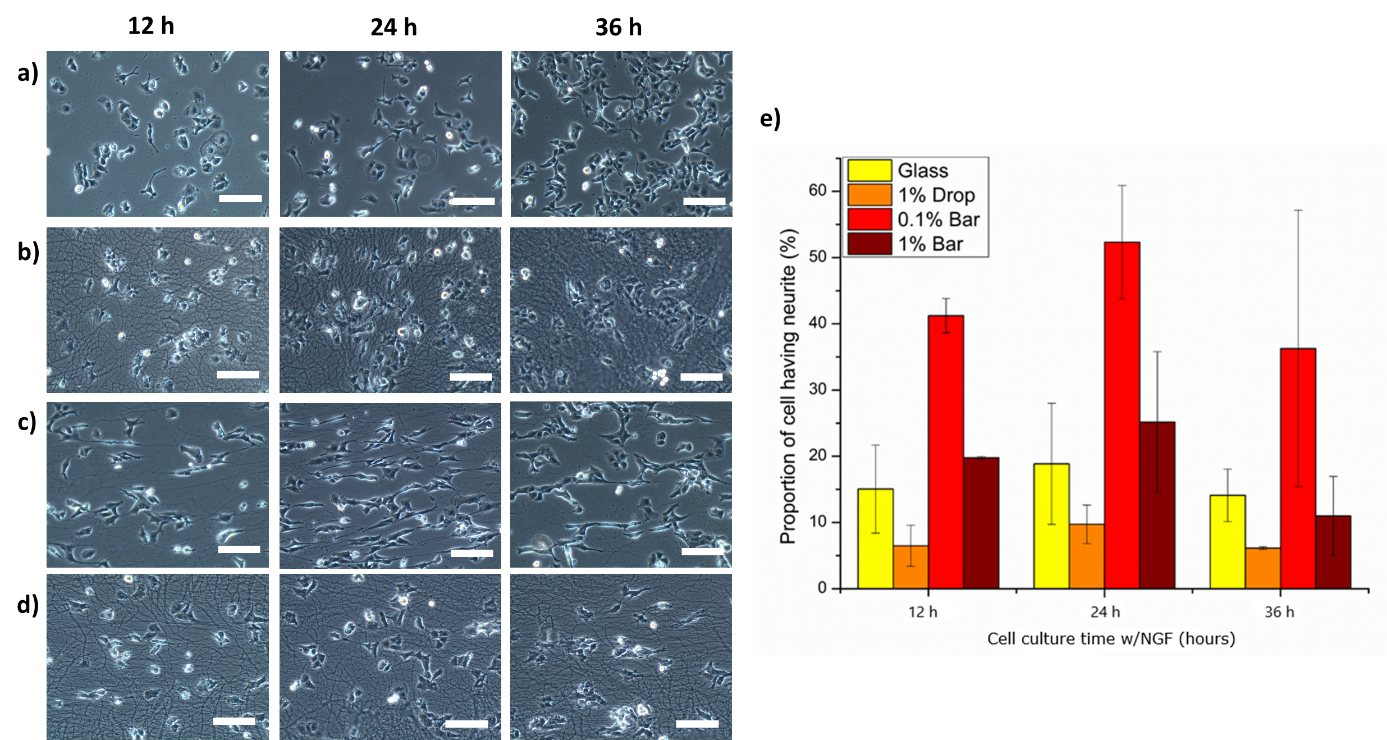


**Figure S6.** PC12 cells cultured in the presence of nerve growth factor (NGF): Optical microscopic images of PC12 cells cultured for 12, 24 and 36 h with NGF on (a) bare glass, (b) 1.0 wt% drop-coated, (c) 0.1 wt% bar-coated, (d) 1.0 wt% bar-coated nFHs. All scale bars denote 100 μm. (e) The percentage of neurite-developed cells (determined by more than 1.5-time longer cell bodies) over total cells on (a) ‒ (d) substrates (n = 200).


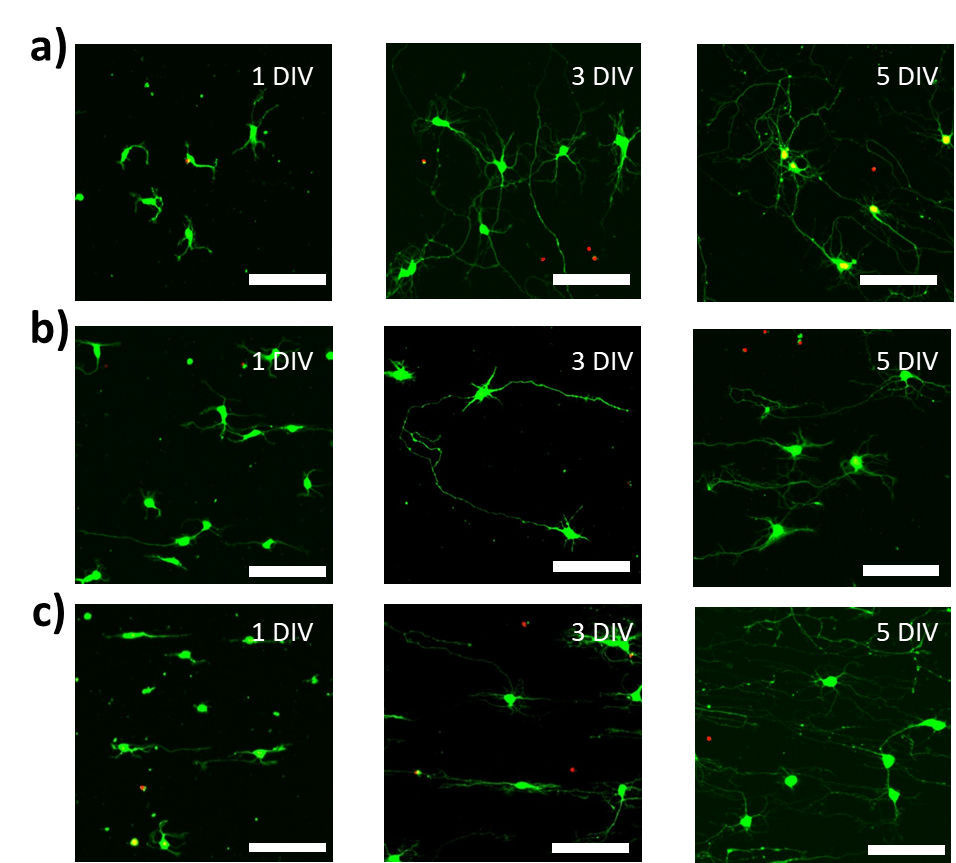


**Figure S7**. Live and dead assay of neuronal cell on a) 1.0 wt% drop-coated glass, b) 0.1 wt% bar-coated glass and c) 1.0 wt% bar-coated glass at 1, 3 and 5 DIV. Green: calcein AM, Red: ethidium homodimer-1 (EthD-1). All scale bars denote 100 μm.


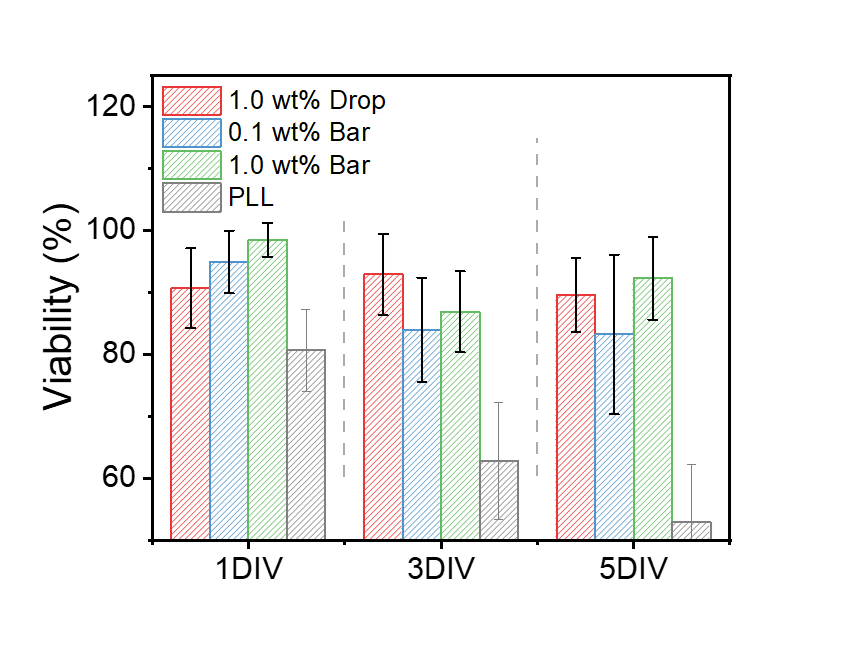


**Figure S8**. Viability of neuronal cells on a nFH-coated and PLL-coated glass substrate at 1, 3 and 5 DIV.
